# Mitigating Membrane Fouling in Abattoir Wastewater Treatment: Integration of Pretreatment Step with Zwitterion Modified Graphene Oxide–Polyethersulfone Composite Membranes

**DOI:** 10.3390/membranes14110227

**Published:** 2024-10-30

**Authors:** Meladi L. Motloutsi, Funeka Matebese, Mxolisi M. Motsa, Muthumuni Managa, Richard M. Moutloali

**Affiliations:** Institute for Nanotechnology and Water Sustainability, College of Science, Engineering and Technology, University of South Africa, Florida Science Campus, Roodepoort 1710, South Africa; 66466393@mylife.unisa.ac.za (M.L.M.); matebf@unisa.ac.za (F.M.); motsamm@unisa.ac.za (M.M.M.); managme@unisa.ac.za (M.M.)

**Keywords:** abattoir wastewater, AEPPS zwitterion, activated carbon, graphene oxide, optimized performance

## Abstract

Composite polyethersulfone (PES) membranes containing N-aminoethyl piperazine propane sulfonate (AEPPS)-modified graphene oxide (GO) were integrated with either of the two pretreatment processes (activated carbon (AC) adsorption or polyelectrolyte coagulation) to assess their effectiveness in mitigating membrane fouling during the treatment of abattoir wastewater. The AEPPS@GO-modified membranes, as compared to the pristine PES membranes, showed improved hydrophilicity, with water uptake increasing from 72 to 118%, surface porosity increasing from 2.34 to 27%, and pure water flux (PWF) increasing from 235 to 673 L.m^−2^h^−1^. The modified membranes presented improved antifouling properties, with the flux recovery ratio (*FRR*) increasing from 59.5 to 93.3%. This study compared the effectiveness of the two pretreatment processes, AC, coagulation, and the integrated system (coagulation/AC-UF membrane), in the removal of natural organic matter (NOM) and improvement of abattoir wastewater’s pH, electrical conductivity, TDS, and turbidity. The integrated systems produced improved water quality in terms of pH, EC, TDS, turbidity, and organic content. The fluorescence excitation–emission matrix (FEEM) analysis exhibited almost no fluorescence peak post-treatment following organic loading removal. The quality of the water met the South African non-potable water reuse standards. The sole membrane treatment systems exhibited good fouling resistance without the pretreatment systems; however, integrating these systems can offer extended longer filtration periods, thereby assisting in cost aspects of the abattoir wastewater treatment system.

## 1. Introduction

Abattoir industries consume enormous amounts of water to maintain high standards of sanitation and hygiene practices during meat processing [1,2]. Abattoir wastewater generated generally has high chemical oxygen demand (COD), biochemical oxygen demand (BOD), suspended solids (SSs), and soluble and insoluble organics. Other contents of this wastewater include undigested food, fat, blood, urine, lint, faeces, flesh pieces, pathogens, etc., that generate sludge deposits to the bottom, scum that floats, increased turbidity, and unbearable odour [2,3,4,5]. Discharging such wastewater directly, or when poorly treated, into nearby rivers or other surface water bodies significantly reduces the quality of receiving water and negatively affects aquatic organisms residing therein [1,6]. It was reported that rivers contaminated with abattoir wastewater have increased levels of metals such as copper, cadmium, chromium, iron, lead, and zinc [6]. Consequently, the treatment and reuse of this type of wastewater for routine equipment maintenance is paramount for environmental considerations and overall water sustainability. Several methods are utilised to treat abattoir wastewater, including combined acoustic cavitation and ozonation [7], the combined activated sludge filtration–ozonation process [8], adsorption [9], bio-coagulation [5,10], electrocoagulation [3], membrane technology [11], submerged anaerobic membrane bioreactor [4,12,13], etc. In order to treat abattoir wastewater to potable water reuse standards, as permitted by national regulations, a treatment system that can remove all toxic contaminants is needed. This is because the conventional treatment system may not be sufficient to upgrade abattoir wastewater to the regulated quality standards.

Membrane technology is extensively used in wastewater treatment due to advantages such as environmental friendliness, selective separation, continuous operation, and energy efficiency [14]. The biological characteristics of abattoir wastewater, such as algae, fungi, bacteria, and protozoa, can be effectively removed through membrane filtration. Although nanofiltration (NF) and reverse osmosis (RO) membranes can effectively remove contaminants from abattoir wastewater and produce high-quality effluent, they are limited in energy consumption and operating costs as they operate at high pressures. Ultrafiltration membranes can also effectively remove contaminants at relatively low costs, and they have demonstrated good reusability properties [15,16,17,18]. Wahyuni et al. [11] reported the removal of COD and BOD from abattoir wastewater using an ultrafiltration membrane. A reduction in COD in treated wastewater is important as it can lead to the depletion of dissolved oxygen in water. The direct use of membranes to treat abattoir wastewater results in rapid deterioration of their efficiency due to fouling through pore blockage and cake formation, amongst other causes [19,20]. Different strategies are used to mitigate the fouling of membranes. These include membrane materials engineering to increase surface hydrophilicity [21] and integration with wastewater pretreatment processes [22]. Activated carbon (AC) is the most used adsorbent in water treatment because it is commercially available, and, thus, it has been widely applied in the hybrid adsorption/UF process. AC has drawn considerable attention in research because of its excellent properties, which include a high surface area, a defined porous structure, high mechanical strength, and many active pores. It is an excellent absorbent that can remove pathogenic microorganisms (bacteria, algae, viruses, protozoa, fungi, etc.) and dissolved organics from wastewater [23,24]. Several studies have reported that AC can significantly adsorb natural organic matter (NOM) and effectively minimise membrane fouling [25,26,27,28,29]. On the other hand, the flocculation process is commonly used because of its greater applicability in the removal of suspended and dissolved solids, colloids, and organic matter found in effluents [30]. The suspended and dissolved solids in wastewater interact with the flocculants to form flocs, which can be easily removed through filtration or sedimentation processes. The flocculation process improves the colour, odour, and turbidity. Therefore, the reduction of suspended and dissolved solids in wastewater assists in reduced membrane fouling [25,31]. A combination of these approaches is known to lead to effective fouling mitigation [32,33], as each process targets specific types of pollutants contained in wastewater.

For instance, membrane engineering to increase surface hydrophilicity can deal with smaller, soluble solutes that passed through the pretreatment step more effectively, while the pretreatment process would have removed pollutants that could have easily blocked the membrane. Materials such as graphene oxide (GO) are commonly employed in the modification of membranes because of their outstanding benefits, which include high chemical stability, low production costs, and high hydrophilicity due to abundant oxygen-containing functional groups, which create more channels for water molecules to travel through. However, GO nanosheets have a stacking tendency over time, which may lead to low permeate flux and poor quality [34,35,36]. Therefore, functionalising GO with organic molecule grafts and polymers will reduce the GO stacking and increase the overall hydrophilic character. Zwitterion-engineered membranes are of interest owing to their unique molecular structures. Zwitterions are characterised by outstanding electrical conductivity, pH responsiveness, a large dipole moment, and abundant oppositely charged functional groups, which result in exceptional hydrophilicity and high polarity. These characteristics bring about a robust hydration layer that hinders foulants from adhering to the membrane surface [37,38]. Previously, we [39] grafted an N-(3-sulfopropyl)-N-(methacryloxyethyl)-N, N-dimethyl ammonium betaine (SBMA) zwitterion onto GO, showing that it diminished the strong π–π and Van der Waals interactions between the GO nanosheets, minimising their stacking tendency. The incorporation of this nanocomposite into the membrane matrix increased the hydrophilicity, permeate flux, and antifouling resistance of the resultant membranes [39]. N-aminoethyl piperazine propane sulfonate (AEPPS), in particular, contains a sulfonate, −SO_3_^−^, functional group, which makes it a highly effective hydrogen bond acceptor and/or contributor. As a result, it may reinforce the mechanism of solute rejection, thus resulting in better membranes. 

In this study, an AEPPS zwitterion was grafted onto GO and blended into the PES matrix for application in abattoir wastewater treatment. In order to mitigate quick membrane fouling and enhance product water quality, membrane filtration was integrated with either AC or coagulation pretreatment steps. The efficiency of the two-pretreatment protocol when integrated with membrane filtration is compared, as well as that without the pretreatment process.

## 2. Materials and Methods

All the materials used in this investigation were purchased from Merck (Johannesburg, South Africa). This included the 1-methyl-2-pyrrolidinone (NMP), N-aminoethyl piperazine (AEP), acetonitrile, 1,3-propanesulfone (1,3-PS), polyethersulfone powder (PES), sulphuric acid (H_2_SO_4_, 98.0%), potassium permanganate (KMnO_4_), hydrogen peroxide (H_2_O_2_, 30%), and hydrochloric acid (HCl ≥ 32%).

### 2.1. Preparation of Graphene Oxide (GO)

The synthesis of GO nanosheets was accomplished using the modified Hummers method [40]. In a 500 mL round-bottom flask containing graphite flakes (3.0 g), H_3_PO_4_ (40 mL) and H_2_SO_4_ (360 mL) were added. This was followed by the slow addition of solid KMnO_4_ (18.0 g) under magnetic stirring. The resultant mixture was heated in an oil bath set to 50 °C for 12 h. The hot solution was allowed to cool to room temperature (RT) and then transferred to 400 mL of crushed ice, followed immediately by the addition of 30% H_2_O_2_ (3 mL). The mixture was later centrifuged (4500 rpm), and the sediments were then rinsed once with deionised (DI) water (200 mL), HCl (30%, 200 mL), two times with ethanol (200 mL), and diethyl ether (200 mL), sequentially, to wash off the unreacted materials and impurities. The product was then air-dried in a fume hood.

### 2.2. Preparation of AEPPS Zwitterion

A ring-opening reaction of 1,3-PS and AEP was used to synthesise N-aminoethyl piperazine propane sulfonate (AEPPS). AEP (6.3 g, 48.76 mmol) was dissolved in acetonitrile (25 mL) in a beaker, while 1,3-PS (6.1 g, 49.94 mmol) was dissolved in acetone (75 mL). The AEP solution was added dropwise into the 1,3-PS solution with stirring at RT in the air. The homogeneous solution was stirred for another 5 h with the formation of a precipitate. The yellow precipitate was collected by filtration and washed with acetone (200 mL). The yellow power was dried overnight at 70 °C (6.2 g (50% yield), 24.67 mmol, AEPPS) [41]. 

### 2.3. Preparation of AEPPS@GO

Benzoyl peroxide (BPO, 5 mL), as an initiator, was added into an aqueous (90 mL) suspension of GO (1.0 g) at 65 °C and further stirred for another 30 min in a 250 mL round-bottom flask. An aqueous (10 mL) solution of AEPPS (0.75 g, 2.98 mmol) was gently added to the GO suspension at 65 °C. The mixture was agitated for another 4 h, centrifuged, and the pellet was washed several times with DI water, then dried overnight at RT to give a black solid (1.030 g) [42].

### 2.4. Fabrication of Membranes

The phase-inversion approach was used to manufacture all flat-sheet membranes [43]. Various amounts of AEPPS@GO (0.1, 0.3, 0.5, 0.7, and 0.9 wt.%) nanocomposites (Table 1) were transferred into a beaker containing N-methyl-2-pyrrolidone (NMP) solvent and swirled for a few minutes at RT. Then, PES powder (18 g) was added to the solution and agitated until it was entirely dissolved. The solutions were stored in a desiccator with the lids tightly closed to allow for the dissipation of gas bubbles. With the help of a 200 µm airgap casting knife, the solution was cast onto a glass plate. The glass with the casting solution was immediately submerged into a coagulation bath containing DI water at RT for film formation. The formed membranes were stored in fresh DI water for 24 h to ensure that all the solvent was removed. Finally, the membranes were kept in DI water at a low temperature until use.

### 2.5. Characterisation of the AEPPS@GO/PES Composite Membranes

The fabricated membranes were first dried in a fume hood before they were characterised using a myriad of techniques. A scanning electron microscope (SEM) (JSM-IT300 Joel, Tokyo, Japan) was used to examine the top surface and cross-sectional morphology of the membranes. The membranes were gold-coated prior to the morphological analysis using a Sputter coater (Quorum Q150R ES, Laughton, UK). Atomic-force microscopy (AFM) (Alpha30 WITec Focus Innovations, Ulm, Germany) was used to estimate the membranes’ quantitative surface roughness. The hydrophilicity of the membrane surfaces was investigated using the sessile water drop to measure the contact angle (CA) (DSA30E Kruss drop shape Analyzer, GmbH, Hamburg, Germany). Fourier transform infrared (FTIR, Perkin Elmer, 100 spectrometers, Karlsruhe, Germany) (PerkinElmer, 100 spectrometers, Germany) was utilised to obtain information on molecular vibrations, which were used to detect different functional groups of the synthesised materials. A fluorescence excitation-emission matrix (FEEM) spectrometer (Aqualog^®^, HORIBA Scientific, Edison, NJ, USA) was used for the ultraviolet–visible absorbance analyses.

DataPhysics Optical Contact Angles (OCA) 15 EC (G10, KRUSS, Hamburg, Germany).

### 2.6. Permeation and Rejection Studies

The dry–wet technique was employed for assessing membrane porosity and water uptake. Membranes with a comparable surface area (A = 0.00126 m^2^) were exposed to DI water for 24 h after first being dried overnight at RT. The weight of the dry membranes was measured before ($M_{dry}$) and after soaking in water for 24 h ($M_{wet}$). The measured cross-section length (*L*) of each membrane, as well as the density of water at the RT (ρ), were used to estimate the water uptake and overall porosity (ε) of the membranes using Equations (1) and (2) [40].

(1) $$Water\;uptake\;\left(\%\right)=\frac{M_{wet}-M_{dry}}{M_{dry}}\times 100$$

(2) $$\varepsilon \left(\%\right)=\frac{M_{wet}-M_{dry}}{\rho_{H_{2}O}\times A\times L}\times 100$$

The diffusion of water across the fabricated membrane (permeability) was investigated using a dead-end cell. Membranes of the same surface area (A) were placed in a dead-end cell, and the cell was filled with DI water, which was pushed through the membranes at different applied pressures. The volume of the collected effluent (*Q*) was measured at 10 min intervals (*t*). The transmembrane pressure (TMP) applied across the membrane ranged between 100 and 500 kPa. Compressed nitrogen gas served as a source of pressure throughout the investigation. The membranes were compacted at a pressure of 700 kPa before assessment. Equation (3) was used for pure water flux (*J_flux_*) calculations.

(3) $$J_{flux}=\frac{Q}{t.A}$$

The ability of the membrane to reject was tested by rejecting three different dyes at 150 kPa, and the UV–Vis spectrometer was used to quantify the concentration of the dye in the permeate and the feed. Equation (4) was applied to determine the dye rejection [44].

(4) $$R=\left(1-\frac{Cp}{Cf}\right)\times 100$$

where *R* symbolises the rejection (*%*), *Cp* symbolises the concentration of the permeate, and *Cf* symbolises the concentration of the feed solution.

For antifouling assessment tests, the membranes were subjected to DI water for 25 min to achieve pure water flux (*J_w_*_,*a*_), followed by filtration of abattoir wastewater for 25 min to obtain flux (*J_p_*). The membranes were then backwashed at a pressure of 400 kPa for 5 min to wash off the foulants followed by pure water filtration for another 25 min to obtain (*J_w_*_,*b*_). The recycled membranes were subjected to pure water and abattoir wastewater filtration again to obtain new flux values. The fouling–washing process was performed for six cycles, which allowed for the determination of the antifouling character of the membranes. The flux recovery ratio (*FRR*), total fouling (*Rt*), reversible fouling (*Rr*), and irreversible fouling (*Rir*) were the fouling metrics that were determined from these assessments and quantified using Equations (5) to (8).

(5) $$FRR\;(\%)=\left(\frac{J_{w,b}}{J_{w,a}}\right)\times 100$$

(6) $$R_{t}\;(\%)=\left(\;1-\;\frac{J_{p}}{J_{w,a}}\right)\times 100$$

(7) $$R_{r}\;(\%)=\left(\;\frac{J_{w,b}-J_{p}}{J_{w,a}}\right)\times 100$$

(8) $$R_{ir\;}(\%)=\left(\frac{J_{w,a}-J_{w,b}}{J_{w,a}}\right)\times 100=R_{t}-R_{r}$$

## 3. Results and Discussion

### 3.1. Characterisation of GO, AEPPS, and AEPPS@GO Nanocomposites

#### 3.1.1. FTIR

**Figure 1:**
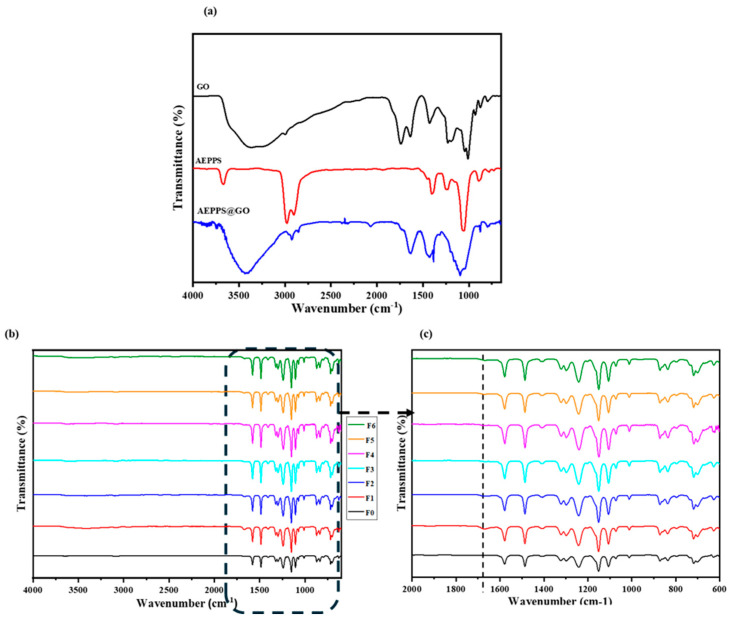
FTIR spectra of (**a**) GO, AEPPS, and AEPPS@GO nanocomposites and (**b**) F0–F6 membranes (**b**,**c**).

FTIR spectroscopy was employed to confirm the change in the intensity of the existing functional groups of the two starting materials, GO and AEPPS, as well as the formation of the new bands in the resultant nanocomposite, AEPPS@GO (Figure 1a). The GO spectrum showed an intense and broad peak assigned to the hydroxyl group, indicating the presence of OH and/or -COOH functional groups at 3400 cm^−1^. The bands observed at 1217 and 1026 cm^−1^ were attributed to C-O-C and C-O stretching of the epoxy and alkoxy groups, respectively, while the carbonyl group (C=O) was observed at ca. 1625 cm^−1^. This is in agreement with the literature [40,45]. For the AEPPS zwitterion, the two bands at 3071 and 3155 cm^−1^ were assigned to the N-H stretching of the primary amide, whilst a band observed at 1037 cm^−1^ is due to the –SO_3_^–^ functional group. Additionally, a band at ca. 1275 cm^−1^ was assigned to the amide C-N functional group [46,47]. The bonding of the GO and AEPPS occurred via the C=O of the GO and the NH_2_ of the AEPPS. This was supported by the C=O band intensity decreasing and the NH_2_ band diminishing in the spectrum of the nanocomposites. These observations provided evidence that AEPPS was successfully grafted onto GO nanosheets. Figure 1a,b (4000–600 cm^−1^) and c (2000–600 cm^−1^) show the FTIR spectra of the fabricated membranes. The pristine PES membrane depicted all the anticipated functional groups of the PES polymer. This includes the stretching adsorption peaks at 1589 and 1294 cm^−1^, which are attributed to the aromatic benzene ring. The symmetric and asymmetric stretching of sulfonate groups were noted at 1103 and 1241 cm^−1^, respectively. The spectra of the modified membranes (GO/PES and AEPPS@GO/PES) retained all the peaks associated with the pristine PES, with the addition of a new, noticeable peak at about 1679 cm^−1^ that was assigned to the carbonyl functionality in GO. 

#### 3.1.2. Contact Angle

**Figure 2:**
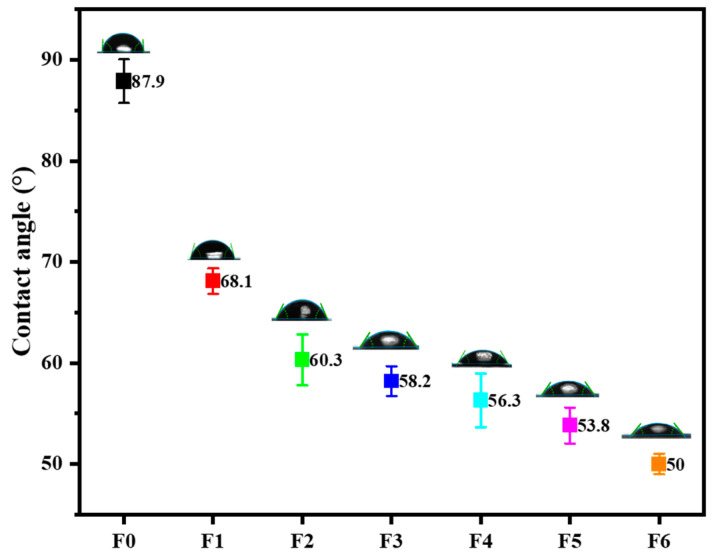
Contact angle results of membranes F0–F6.

The surface hydrophilicity of the prepared membranes was investigated using the contact angle (CA), and the results are illustrated in Figure 2. The pristine PES membrane exhibited the highest CA of 87°, indicative of its relative hydrophobic nature, in line with prior reports [48]. The CA was reduced to 69° upon the addition of GO, indicating improved hydrophilicity due to the presence of the hydrophilic filler provided by the oxygen-containing functional groups of GO. A further decrease in CA was observed with the addition of the composite (AEPPS@GO) into the membrane matrix, indicating higher hydrophilicity contributed by the AEPPS@GO nanofillers. The CA ranged from 60 to 53°, showing a decreasing trend as the AEPPS content increased in the composite. These observations were in line with the literature [22,29].

#### 3.1.3. Water Uptake and Porosity Measurements

**Figure 3:**
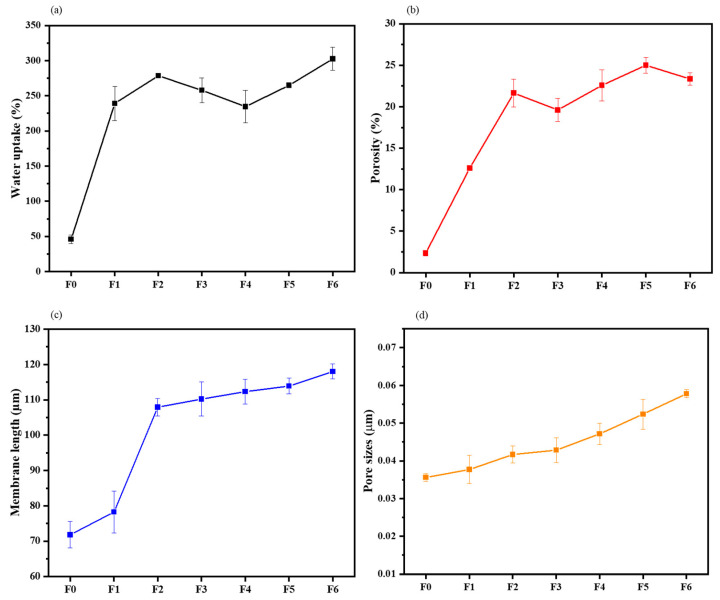
(**a**) Water uptake, (**b**) bulk porosity, (**c**) membrane’s cross-sectional length, and (**d**) average pore size distribution of membranes F0–F6.

The water uptake and porosity of the membranes were determined in order to study the structural effects of incorporating GO and AEPPS@GO (Figure 3a,b). The membrane’s thickness and pore sizes were also determined, as they play a role in the capabilities of water uptake and porosity. The water uptake capability of the pristine membrane was found to be 72%, which then increased to 78% upon the introduction of GO. There was a further gradual increase, congruent with the increasing AEPPS content from 108 to 118% for the AEPPS@GO/PES membranes. For porosity, the pristine PES membrane presented a porosity of 2.34%. Following the modification of the membrane with hydrophilic GO, an increased porosity of 12% was noted. The porosity increased further, to between 20 and 27%, after the incorporation of the AEPPS@GO composites. The incorporation of GO and AEPPS has helped to increase the water channels of the membranes and the water-loving groups, which helped with water absorption. The cross-section length of the membranes (Figure 3c) was measured prior to and post-modification. A slight increase from 72 to 78 µm was observed after the inclusion of GO in the PES matrix. A larger increase from 108 to 118 µm was observed after the incorporation of AEPPS@GO in the membranes. Therefore, length gradually increased with an increase in AEPPS content in the AEPPS@GO composite. The surface pore sizes, measured from the SEM micrographs using ImageJ, followed a similar trend (Figure 3d), that is, increasing slightly as the AEPPS content increased. The surface pore sizes increased from 0.035 µm for F0 to 0.057 µm for the F6 membrane. The enhancement in these properties can be attributed to the faster demixing process facilitated by GO and AEPSS as a result of their higher hydrophilicity. This is in line with the literature [49,50].

#### 3.1.4. AFM

**Figure 4:**
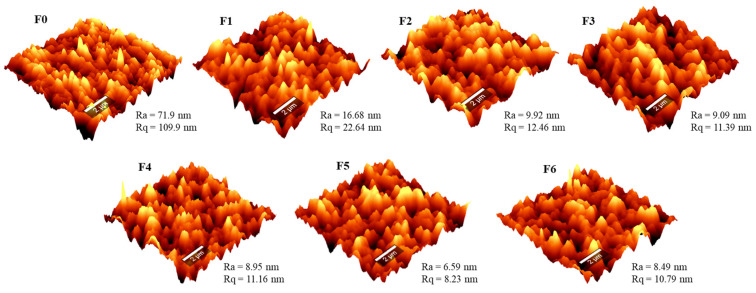
AFM micrographs showing the surface roughness of membranes F0–F6.

The surface roughness parameters of the membranes were evaluated using AFM analysis. Figure 4 presents AFM images, *Ra* (mean roughness), and *Rq* (root mean square) parameters. The pristine PES membrane exhibited a surface roughness of 71.9 nm, and this was due to the hydrophobic nature of PES, resulting in a delayed solvent and non-solvent demixing rate. Following the modification of the PES membrane with GO, the roughness parameter drastically decreased to 16.68 nm. The decrease was expected and in line with the reported literature trends; the addition of hydrophilic functional groups to the surface reduces the roughness of the membrane and, hence, its fouling propensity [51]. The AEPPS@GO/PES membranes exhibited lower roughness parameters, ranging between 6.56 and 9.92 nm. The addition of the hydrophilic function group from the AEPPS@GO nanofiller resulted in a smoother membrane surface.

#### 3.1.5. SEM

**Figure 5:**
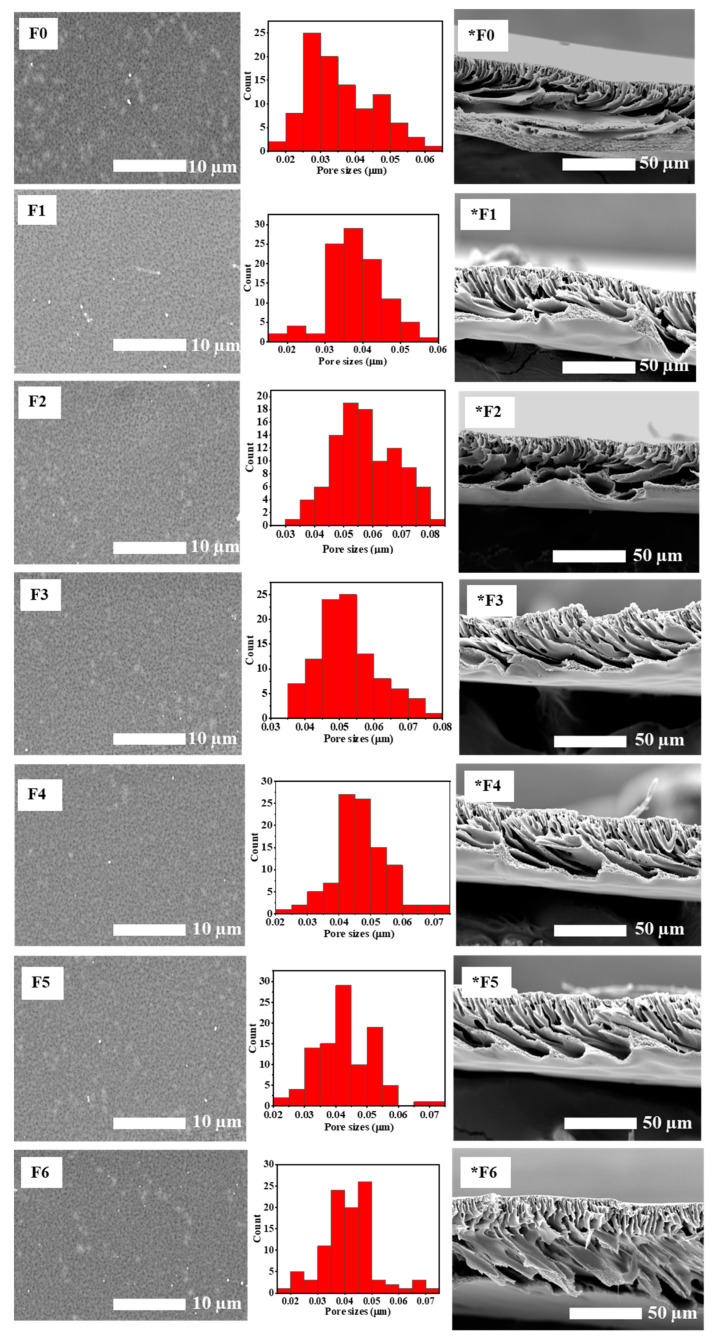
SEM micrographs showing the top surface, pore size distribution, and cross-section (*) of F0–F6 membranes.

The top surface, cross-sectional morphologies, and the pore size histograms, of the membranes are presented in Figure 5. All the membranes presented a porous surface with pores of varying sizes ranging from 0.035 to 0.057 µm. The sizes of the membrane pores (Figure 3d) increased in the order PES<GO/PES<AEPPS@GO/PES. The pore size distribution depicted an increasing trend in pore size upon an increase in AEPPS concentration. The average pore sizes of the pristine PES membrane were found to be 0.035 µm and 0.037 µm for GO/PES and to range between 0.041 and 0.057 µm for the AEPPS@GO/PES membranes. Furthermore, an increasing trend of pore sizes was observed with an increased loading of AEPPS@GO nanocomposites. The cross-section of the membranes exhibited an asymmetric structure characterised by a thin top layer and finger-like macro-voids typical of UF membranes produced by the NIPS method [52]. An increase in the macro-void size was observed in the matrix of the membranes due to the incorporation of the hydrophilic GO. The hydrophilicity of GO and AEPPS@GO contributed to a fast water and solvent exchange during the fabrication process, resulting in the observed structure [53]. These findings are in line with the reported literature [42,54].

### 3.2. Membrane Application

The pristine PES, GO/PES, and AEPPS@GO/PES composite membranes were assessed, and all the data obtained are presented below.

#### 3.2.1. Pure Water Flux (PWF)

**Figure 6:**
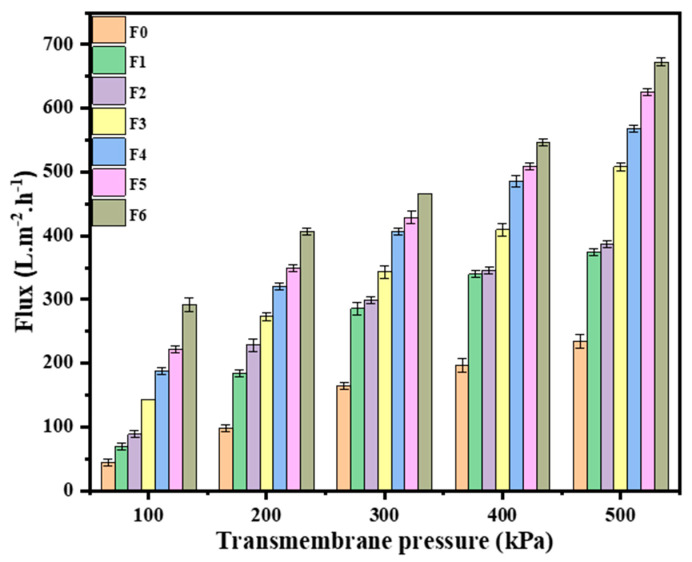
The pure water fluxes of the pristine PES (F0), GO/PES (F1), and AEPPS@GO/PES (F2–F6) at different loadings of AEPPS.

The PWF (Figure 6) of the pristine PES membrane ranged from 44 to 235 L.m^−2^.h^−1^ from the lowest to the highest transmembrane pressure. The GO/PES membranes presented an improved PWF from 70 to 375 L.m^−2^.h^−1^ compared to that of the pristine PES membrane. This was attributed to the effect of the introduction of GO, as previously reported in the literature [21]. GO is reported to impart a hydrophilic character to the membranes as well as create additional water channels; both of these properties aid in water passage [40]. An increase from 89 to 417.31 L.m^−2^.h^−1^ was observed after the incorporation of 0.1 wt.% AEPPS@GO onto the PES membrane. Further improvements were observed after the blending of AEPPS@GO (0.3, 0.5, 0.7, and 0.9 wt.%) onto PES. This was expected since both AEPPS and GO have hydrophilicity-improving functional groups that enhance the rate of water passing through the membrane at a given time [55].

#### 3.2.2. Relative Fouling Propensity and Reusability Tests of Membranes

**Figure 7:**
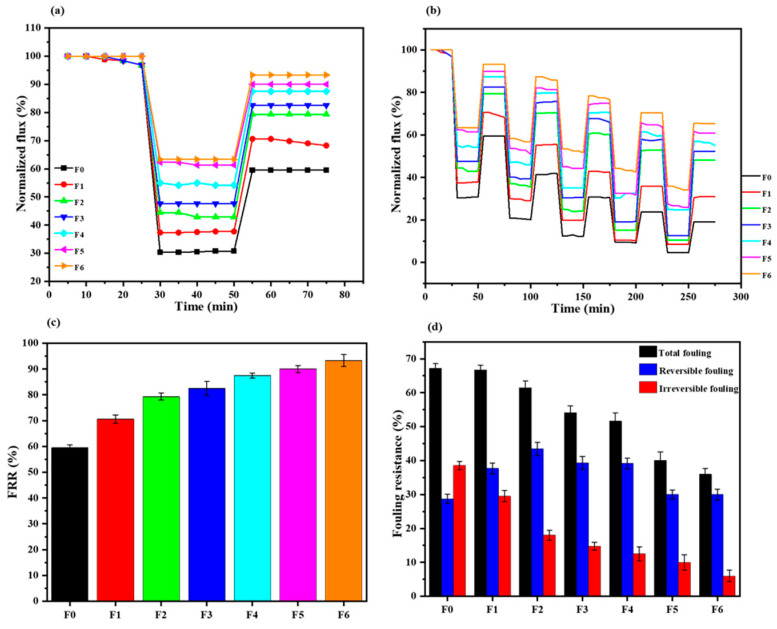
Flux versus time of prepared membranes during the fouling process (**a**) single cycle and (**b**) multiple cycles. (**c**) Flux recovery ratio and (**d**) fouling resistance parameters of the fabricated membranes (F0–F6).

The antifouling behaviour of the fabricated membranes was evaluated using abattoir wastewater. In Figure 7a, a sharp decrease in flux was noted when filtering the wastewater (30–50 min), suggesting the accumulation of pollutants from the abattoir wastewater on the membrane surface or within the pores. After backwashing, the membranes recovered the fluxes as pure water filtration was higher. The rate at which the membranes fouled and recovered depended on the membrane type. The modified membranes, as expected, showed better performance owing to their engineered surface. Over a single cycle (Figure 7c), the flux recovery ratio (*FRR*) follows the increasing AEPPS content, with the top two membranes exhibiting an *FRR* of over 90%. In fact, all membranes containing AEPPS exhibited *FRRs* above 79%, whilst those containing only GO were at 70%. The pristine PES membrane showed the lowest *FRR* of just above 59.5%. A similar trend was observed over the six fouling–washing cycles (Figure 7b). In this case, the top four (containing high AEPPS@GO content) performing membranes exhibited over 50% of the *FRR* after the six cycles. The membrane with the least amount of AEPPS@GO, as well as the one containing GO, only showed *FRRs* of 48 and 30.8%, respectively. The pristine PES membrane could only exhibit about 19% of the *FRR*. These observations indicated that membranes containing AEPPS@GO nanofillers possessed higher antifouling characteristics and good reusability potential for abattoir wastewater treatment. The fouling resistance was further interrogated in-depth using three fouling parameters, i.e., total fouling (*Rt*), reversible fouling (*Rr*), and irreversible fouling (*Rir*) ratios, and their percentages were calculated to give insight into foulant interaction with the membrane surface (Figure 7d). The pristine PES membranes presented an *Rt* of 67.2%, of which 38.5% was irreversible and only 28.7% reversible. These results indicated that the pristine PES membrane was prone to fouling that was difficult to reverse, which could be attributed to its relatively high surface roughness and relatively hydrophobicity. The GO/PES membrane, on the other hand, presented a slightly lower *Rt* of 66.7%, with *Rr* of 37.7% and *Rir* of 29%. The AEPPS@GO/PES membranes exhibited even better antifouling behaviours. For instance, the membrane with the least amount of AEPPS presented *Rt*, *Rr*, and *Rir* of <62%, 43.4, and 18.0%, respectively. Furthermore, these parameters continued to improve as AEPPS loading increased; the F6 membrane exhibited the lowest *Rt*, *Rr*, and *Rir* of 36.6, 30, and 6.6%, respectively. The improving fouling resistance with the addition of GO and AEPPS@GO nanofillers was observed to increase with an increase in surface smoothness and hydrophilicity, both of which are known to mitigate membrane fouling [56,57]. The modification with GO and the zwitterion was expected to increase the fouling resistance due to the presence of a hydration layer from zwitterions that minimises foulant interaction with the membrane surface as well as abundant oxygen-containing functional groups from GO that enhance hydrophilicity. Therefore, the membranes containing GO and AEPPS@GO were expected to perform better than the pristine membranes, a scenario that was reported [55]. Furthermore, mechanistic interrogation of this conundrum might shine a light on the underlying phenomenon. It is also noted that surface roughness decreased with increasing AEPPS content in the nanofiller, which might also contribute to the observed increase in fouling resistances, as previously reported [38] and observed earlier (Figure 4).

#### 3.2.3. Characteristics and Quality of Abattoir Wastewater

The performance of the fabricated membranes was assessed using abattoir wastewater. The treatment efficiency was monitored by measuring pH, turbidity, total dissolved solids (TDSs), electrical conductivity (EC), and salinity before and after treatment. Furthermore, to mitigate membrane fouling by the abattoir wastewater, a pretreatment step was incorporated whereby chemical flocculation or AC was performed prior to membrane filtration. Therefore, the quality of the wastewater was tested after coagulation or AC pretreatment as well as after coagulation/AC ultrafiltration (Table 2 and Table 3), respectively. This study was investigated using the three best-performing membranes (F4, F5, and F6) and reference membranes (F0 and F1). Integrating the pretreatment (coagulation or AC) and UF membrane systems guarantees effluent that can be discharged into the environment or reused in abattoir processing.

Table 2 shows abattoir wastewater received without pretreatment from the abattoir house, then pretreated with AC, and followed by the fabricated UF membranes in the lab. The AC pretreatment improved the feed by 25 and 31% for TDS and turbidity, respectively. An improvement in pH, EC, and salinity was also observed. The odour also subsided after AC pretreatment. The base membranes exhibited a slight improvement in TDS, pH, EC, and salinity, while, in turbidity, a significant improvement of 94.2 and 96.1% for F0 and F1 was observed. The turbidity of the AC–UF-treated water further decreased to 98% for the F5 membrane. Table 3 shows the abattoir wastewater pretreated at the abattoir house with coagulants, followed by the use of fabricated UF membranes for the high removal of contaminants. The base membranes (F0 and F1) improved TDS to 45 and 47%, while significantly improving turbidity to 96 and 98%, respectively. The best-performing membrane, F5, improved TDS and turbidity by 52 and 99.8%, respectively. The AC–F5 and AC–F6 and coagulant–F5 and coagulant–F6 treatment systems showed better performance than all other treatment systems, and this can be attributed to their high antifouling properties. The coagulant–membrane treatment system was more effective for abattoir wastewater since it exhibited lower TDS and turbidity compared to the AC–membrane treatment system. The salinity of the coagulant–UF systems improved to 0.02 ppm compared to 0.03 ppm in the AC–UF systems. Utilising coagulants can generate less sludge compared to AC, and the economic costs are much lower in view of the fact that small amounts are used for the coagulation process [58,59]. The quality of the abattoir effluent was upgraded to below the minimum standards (SANS 241:2015) for discharge limits for pH, TDS, EC, and turbidity for both the coagulant–membrane and AC–membrane treatment systems. The AEPPS@GO membrane integrated systems exhibited better performance due to high hydrophilicity (Figure 2 and Figure 3), smoother surfaces (Figure 4), high permeability (Figure 6), and relatively excellent antifouling properties (Figure 7). Additionally, UF membrane size exclusion mechanisms can effectively remove biological and chemical characteristics, which include COD, BOD, organic acids, etc., from abattoir wastewater [30,60].

#### 3.2.4. FEEM Analysis

**Figure 8:**
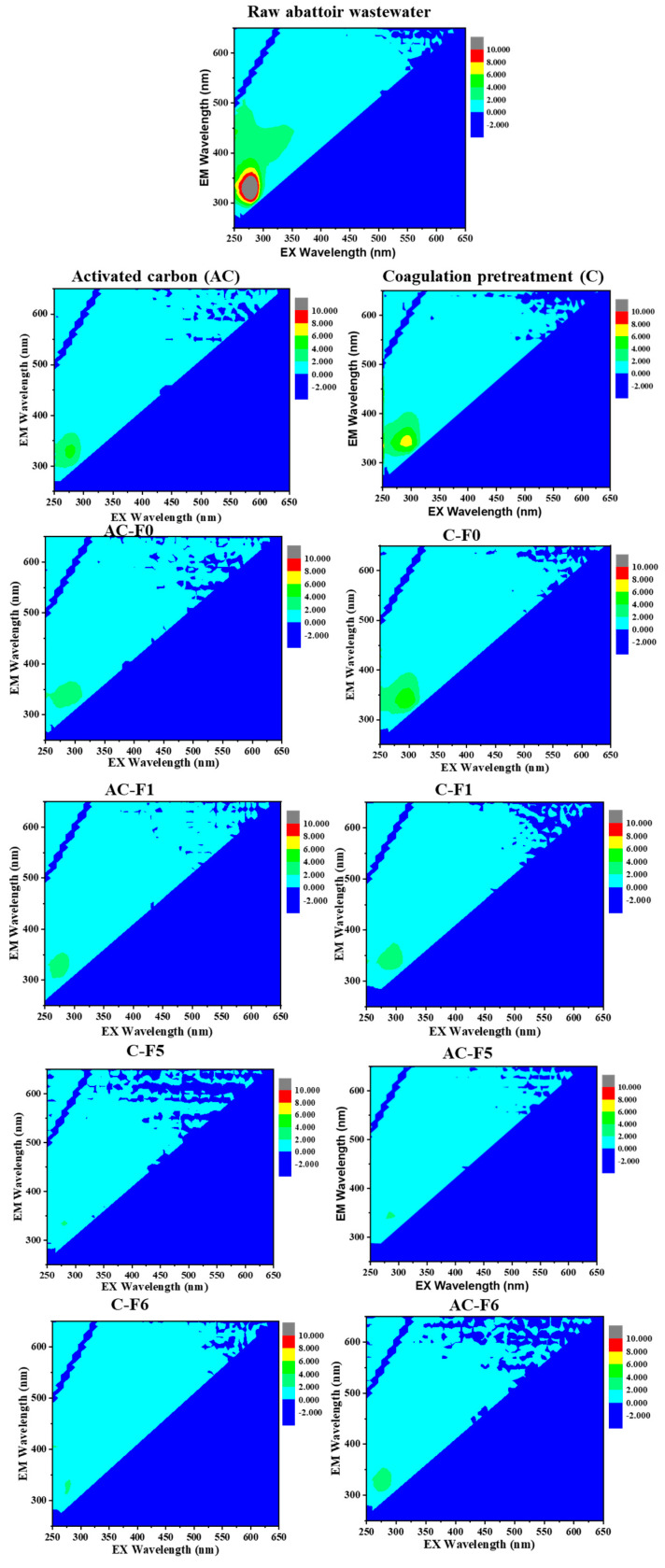
FEEM plots of raw abattoir wastewater, pretreated water, and integrated system treated water.

The three-dimensional (3D) fluorescence excitation emission matrix (FEEM) was employed to monitor the organic characteristics of the abattoir wastewater using two different integrated systems (the coagulation–UF system and the AC–UF system) (Figure 8). According to the work reported by Chen et al. [61], the excitation and emission wavelength boundaries are defined into five regions for different organic substances. These organic substances are grouped and described into five classifications: (I) tryptophan-like proteins (λ_EX/EM_ = 220–250/280–330 nm); (II) tyrosine-like proteins (λ_EX/EM_ = 220–250/330–380 nm); (III) fulvic acid-like substances (λ_EX/EM_ = 220–250/380–480 nm); (IV) soluble microbial product substances (λ_EX/EM_ = 220–350/280–380 nm); and (V) humic acid-like substances (λ_EX/EM_ = 220–450/230–560 nm). Three-dimensional fluorescence spectra of the raw water, pretreated water, and the ultrafiltration system are shown in Figure 8. The results on raw abattoir wastewater using the 3D-FEEM outcomes exhibited the strongest fluorescence peak in the II to IV regions, indicative of the presence of organic substances such as tyrosine-like proteins, fulvic acid-like compounds, and soluble microbial products. The darker the shade, the higher the fluorescence intensity in that region. After treatment with coagulation and adsorption with activated carbon, the response in the fluorescence in regions II and IV was significantly reduced. A significant decrease in the fluorescence peak was observed more in AC relative to coagulation, implying that AC had an obvious removal effect on the organic matter in the abattoir wastewater. A decrease in the fluorescence peak was observed after integrating the pristine PES membrane. A further decrease in the fluorescence peak was observed upon treatment with the GO/PES membrane. The AEPPS@GO/PES membranes (for C-F6 and AC-F6) exhibited almost no fluorescence peaks, which was attributed to the outstanding effectiveness of the nanofillers incorporated into the PES membrane.

## 4. Conclusions

This study describes the synthesis of AEPPS@GO nanofillers and their incorporation into the PES matrix in the fabrication of AEPPS@GO/PES membranes (F0-F6) and their characterisation. The performance of the prepared membranes was investigated relative to the pristine PES membrane. The modified membranes showed improved surface characteristics such as surface hydrophilicity and smoothness. These characteristics enhanced the membrane performance, like PWF and fouling resistance. The membranes containing GO (GO/PES) and AEPPS@GO (AEPPS@GO/PES) presented higher PWF values relative to the pristine PES membrane under the same condition, which increased with trans-membrane pressure. The AEPPS@GO/PES membranes presented an *FRR* of between 73% and 93.3%, maintaining relatively good performance over the six fouling–washing cycles compared to the baseline membranes (PES (59.5%) and GO/PES (70.6%)). Furthermore, these membranes were integrated with either AC or coagulation pretreatment for the removal of organic characteristics from abattoir wastewater, and the efficiency of the integrated systems was compared. The FEEM plots exhibited almost no fluorescence peaks after using the integrated systems, indicating the good properties of activated carbon and coagulation. The coagulation–UF systems presented better results relative to the AC–UF systems based on the quality of the final effluent, i.e., EC, turbidity, TDS, and salinity. On the other hand, the AC-UF filtration system showed better removal of organic characteristics as observed from the FEEM results, a result attributed to its known ability in the removal of organics. The treated abattoir water quality using both integrated systems was improved to comply with the minimal discharge requirements (SANS 241:2015) for discharge with regard to turbidity, pH, TDS, and EC. Thus, the integrated systems tested in this work have a potential for real application in abattoir industries. These integrated systems offer affordable and effective alternatives when compared to other treatment systems, such as NF and RO, based on costs.

## Figures and Tables

**Table 1:** PES, GO/PES, and AEPPS@GO/PES casting solution compositions.

Membrane ID	PES (wt.%)	GO (wt.%)	AEPPS@GO (wt.%)	NMP (wt.%)
F0	18	-	-	82
F1	18	0.5	-	81.5
F2	18	-	0.1	81.9
F3	18	-	0.3	81.7
F4	18	-	0.5	81.5
F5	18	-	0.7	81.3
F6	18	-	0.9	81.1

**Table 2:** Raw abattoir water pretreated with AC and then UF membranes.

Sample ID	pH	Conductivity (µS.cm^−1^)	TDS (mg/L)	Turbidity (NTU)	Salinity (ppm)
Standards	5.5–9.5	≤170	≤1200	≤5	1000
Feed	6.25	1.81	901	55.2	0.12
AC	6.80	1.44	674	38.1	0.6
F0	7.41	1.41	669	3.21	0.03
F1	7.59	1.37	644	2.14	0.03
F4	7.63	1.36	640	1.82	0.03
F5	7.77	1.30	456	1.15	0.03
F6	7.69	1.34	609	1.60	0.03

**Table 3:** Abattoir water pretreated with coagulation and then ultrafiltration membranes.

Sample ID	pH	Conductivity (µS.cm^−1^)	TDS (mg/L)	Turbidity (NTU)	Salinity (ppm)
Standards	5.5–9.5	≤170	≤1200	≤5	1000
Feed	6.25	1.81	901	55.2	0.12
Coagulant	6.40	1.08	501	31.0	0.6
F0	6.95	1.03	489	2.25	0.02
F1	7.13	0.99	472	1.20	0.02
F4	7.17	0.85	463	0.84	0.02
F5	7.38	0.77	432	0.12	0.02
F6	7.35	0.80	456	0.26	0.02

## Data Availability

The raw/processed data required to reproduce these findings cannot be shared at this time as the data also form part of an ongoing study.
